# Candidate prioritization for low-abundant differentially expressed proteins in 2D-DIGE datasets

**DOI:** 10.1186/s12859-015-0455-x

**Published:** 2015-01-28

**Authors:** Umesh K Nandal, Wytze J Vlietstra, Carsten Byrman, Rienk E Jeeninga, Jeffrey H Ringrose, Antoine HC van Kampen, Dave Speijer, Perry D Moerland

**Affiliations:** 1 Bioinformatics Laboratory, Academic Medical Center, University of Amsterdam, PO Box 22700, DE Amsterdam, 1100 The Netherlands; 2 Laboratory of Experimental Virology, Department of Medical Microbiology, Center for Infection and Immunity Amsterdam (CINIMA), Academic Medical Center, University of Amsterdam, PO Box 22700, DE Amsterdam, 1100 The Netherlands; 3 Biosystems Data Analysis Group, University of Amsterdam, Science Park 9041098, XH Amsterdam, The Netherlands; 4 Department of Medical Biochemistry, Academic Medical Center, University of Amsterdam, PO Box 22700, DE Amsterdam, 1100 The Netherlands

**Keywords:** Prioritization, Proteomics, 2D-DIGE, Network

## Abstract

**Background:**

Two-dimensional differential gel electrophoresis (2D-DIGE) provides a powerful technique to separate proteins on their isoelectric point and apparent molecular mass and quantify changes in protein expression. Abundantly available proteins in spots can be identified using mass spectrometry-based approaches. However, identification is often not possible for low-abundant proteins.

**Results:**

We present a novel computational approach to prioritize candidate proteins for unidentified spots. Our approach exploits noisy information on the isoelectric point and apparent molecular mass of a protein spot in combination with functional similarities of candidate proteins to already identified proteins to select and rank candidates. We evaluated our method on a 2D-DIGE dataset comparing protein expression in uninfected and HIV-1 infected T-cells. Using leave-one-out cross-validation, we show that the true-positive rate for the top-5 ranked proteins is 43.8%.

**Conclusions:**

Our approach shows good performance on a 2D-DIGE dataset comparing protein expression in uninfected and HIV-1 infected T-cells. We expect our method to be highly useful in (re-)mining other 2D-DIGE experiments in which especially the low-abundant protein spots remain to be identified.

**Electronic supplementary material:**

The online version of this article (doi:10.1186/s12859-015-0455-x) contains supplementary material, which is available to authorized users.

## Background

Identification of proteins, their posttranslational modifications, and quantification of their abundance is essential for understanding cellular processes, such as the cellular response to virus infection [1-3]. A frequently used technique for measuring protein abundance is 2D gel electrophoresis (2DE). In 2DE a complex protein mixture is separated both on isoelectric point (pI), using isoelectric focusing, and apparent molecular mass (Mw). Based on these two properties, proteins migrate to different locations on a gel and their abundance can be estimated from staining or, upon prior labeling, from the amount of fluorescence. 2DE is very often combined with mass spectrometry (MS) to identify proteins excised from spots on the gel. To decrease gel-to-gel variation and increase sensitivity, two-dimensional differential gel electrophoresis (2D-DIGE) was developed. 2D-DIGE enables quantification of changes in protein abundance by fluorescent labelling of samples with Cy3 or Cy5 and running these on the same gel. Quantification is improved even further by repeating experiments (using biological replicates) and using a Cy2-labeled internal standard consisting of a pool of equal amounts from all samples investigated in the experiment [4]. However, reliable identification of low-abundant proteins after 2D-DIGE is still challenging. Crucial in this respect is that fluorescent labeling by Cy3 or Cy5 is over 40-fold more sensitive than the most sensitive silver stain [5]. As a consequence low-abundant differentially expressed proteins do not become available for follow-up mass-spectrometric analysis upon colloidal Coomassie restaining of the gel. Indeed, often more than half of the differentially expressed protein spots cannot be identified using peptide mass fingerprinting, in combination with either matrix assisted laser-desorption time of flight (MALDI-TOF) or liquid chromatography (LC)-MS/MS analysis, due to the scarcity of the protein they contain [6,7].

Recently, several computational approaches have been proposed that enhance protein identification by exploiting information about the biological context relevant to the performed experiment [8]. Gwinner et al. [9] developed a method to generate a list of candidate proteins that might have remained undetected in a 2D-DIGE experiment. Their approach involves the construction of a Steiner tree on a protein-protein interaction network, which connects already identified, differentially expressed proteins. The nodes of the Steiner tree form a set of suitable candidate proteins that can be validated using Western blotting, for example. Protein differences in the low-abundant range are also difficult to detect using the newest (gel-free) shotgun LC-MS/MS techniques. All proteomic MS analyses are hampered by well known dynamic range problems, in which the most abundant protein around ‘sets’ the limit of detection for the experiment. Network-based approaches have therefore also been proposed to (re-) mine MS/MS experiments in order to increase protein identification. Ramakrishnan et al. [10] used a diffusion algorithm to propagate the evidence from an MS experiment along the edges of a yeast gene functional network. Proteins that did not pass the confidence threshold for identification can be rescued if proteins in their network neighbourhood were reliably identified. Li and colleagues [11] used protein interaction networks to search for cliques, that is, completely connected subnetworks. A low-confidence protein is rescued if it is a member of a clique that is enriched for reliably identified proteins. Whereas these two approaches do not use quantitative information for protein identification, such information can also be exploited. SNIPE [12] uses a network-based approach in which the spectral counts of a protein and its direct neighbours in a functional network are combined. In a case-control experiment the resulting counts can then be used to highlight proteins that are likely to be active but not detectable in a shotgun proteomic experiment.

In this paper, we present a novel computational approach to prioritize candidate proteins for unidentified low-abundant (non-stainable) spots in 2D-DIGE experiments. A limitation of the Steiner tree approach of Gwinner et al. [9] mentioned above is that additional information available for each unidentified spot, namely the pI and Mw of the protein(s) that migrated there, is completely ignored. Our prioritization approach specifically exploits this information in order to propose a list of candidate proteins for each unidentified spot. Functional similarities of candidate proteins to already identified proteins are then used to rank candidates. We applied our prioritization approach to protein spots differentially expressed at the peak of HIV-1 infection of CD4^+^ T-cells [6]. The procedure developed here shows promise for (re-)mining 2D-DIGE datasets and for obtaining insights regarding expression differences of low-abundant proteins that cannot (yet) be found using alternative methods.

## Methods

### 2D-DIGE data

The dataset used in this study was generated in a 2D-DIGE proteomic experiment comparing uninfected and HIV-1 infected PM1 T-cells [6]. First-dimension isoelectric focusing (IEF) of the samples was performed using 24-cm precast immobilized pH gradient (IPG) strips (pH 3 to 11, nonlinear [NL]; GE Healthcare). Next, second dimension sodium dodecyl sulfate polyacrylamide gel electrophoresis (SDS-PAGE) was performed. After image acquisition and analysis, 296 significantly differentially expressed protein spots were detected at 7-10 days post infection. Performing peptide mass fingerprinting (PMF) with a MALDI-TOF mass spectrometer, 93 unique proteins were identified from 108 spots. UniProt IDs of the identified proteins were updated and three spots corresponding to protein fragments were left out, leading to 105 spots corresponding to 92 unique proteins. See Additional file 1 for the complete list of PMF-identified proteins and their characteristics. The remaining 188 spots did not contain enough protein to allow identification by PMF.

### Candidate protein prioritization

**Figure 1:**
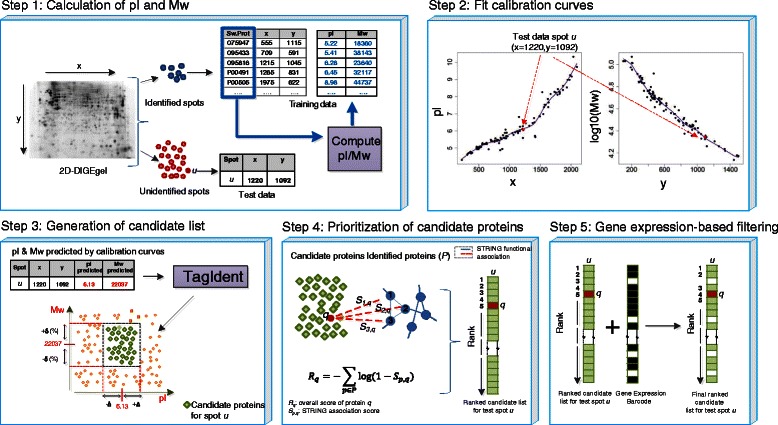
**Prioritization of candidate proteins based on pI and Mw.** Step 1: pI and Mw (Da) of the mature forms of the proteins identified by PMF are determined using the ExPASy tool “Compute pI/Mw” [13]. Step 2: The (*x*,*y*) coordinates of the identified spots and their corresponding pI and Mw (on log10-scale) are used as training data for fitting two cubic smoothing splines. Step 3: For an unidentified test spot *u*, a candidate list of proteins is generated using the ExPASy tool TagIdent [14] by specifying ranges *Δ* and *δ*(*%*) around the pI and Mw predicted by the smoothing splines, respectively. Step 4: Proteins in the candidate list are ranked by calculating their similarities with the PMF-identified ‘seed’ proteins using STRING association scores. Step 5 (optional): The ranked candidate list can be further filtered using presence (black) and absence (white) calls from the Gene Expression Barcode 3.0 [15]. A protein is excluded from the ranked list if the corresponding gene is expressed on none of the selected microarrays.

The objective of our method is to identify the most likely protein candidates for low-abundant differentially expressed spots. Our method uses proteins identified by PMF and their (*x*,*y*) coordinates on the gel to prioritize candidate proteins for unidentified spots. In this section we present the different steps of our prioritization approach (Figure 1).

**1. Calculation of pI and Mw.** In most cases the specific isoform detected on a gel represents the most abundant, mature protein form. We compute theoretical pI and average Mw for the mature form of the PMF-identified proteins using “Compute pI/Mw” [16] given their UniProt protein accession numbers. We added 42 Da to the Mw of those proteins for which N-terminal acetylation was detected by mass-spectrometric analysis of the digested protein spot.

**2. Fit calibration curves.** Both for first dimension IEF using IPG strips and SDS-PAGE, standard protocols generate gels with well-characterized profiles. IPG strips are available that show either linear or smooth non-linear profiles across a specified pH range. SDS-PAGE separation is characterized by an approximately linear relationship between the logarithm of Mw and the migration distance. In most cases the extremes of the pI and Mw ranges are less well-defined and show smooth non-linear gradients. We therefore fitted two cubic smoothing splines for pI and Mw (on log10-scale), respectively, using the R function *smooth.spline*. The pI fit is estimated from the *x*-coordinates of the identified spots and the corresponding theoretical pI as determined in the previous step. The Mw fit is estimated from the *y*-coordinates of the identified spots and the corresponding average Mw (on log10-scale). Optimal values for the smoothing parameter are determined using generalized leave-one-out cross-validation.

**3. Generation of candidate list.** We use the two calibration curves to predict the pI and Mw for unidentified protein spots from the corresponding (*x*,*y*) coordinates. From the estimated pI and Mw a list of candidate proteins is then generated using TagIdent [16] by specifying the pI range (*Δ*) and Mw range (*δ*) in which the search has to take place. Candidate proteins are retrieved from all human proteins contained in UniProtKB/Swiss-Prot.

**4. Prioritization of candidate proteins.** Candidate lists generated in the previous step often contain hundreds of proteins. We prioritize candidate proteins based on their similarity to the set of PMF-identified ‘seed’ proteins *P* using the principle of guilt by association [17]. In our prioritization approach we use functional protein-protein associatons provided by the Search Tool for the Retrieval of Interacting Genes (STRING) database (version 9.1) [18]. For a given pair of proteins (*p*,*q*), STRING integrates evidence from multiple sources such as co-occurrence in pathways, physical protein-protein interaction, co-occurrence in the abstracts of scientific reports etc., and provides a probabilistic score 0≤*S*_*p*,*q*_<1 for the strength of association. For each candidate protein, we determine an overall score by combining the association scores *S*_*p*,*q*_ of a candidate protein *q* and the PMF-identified proteins *p*∈*P* as follows:

(1) $$ R_{q} = - \sum_{p \in P} \text{log}(1-S_{p,q})  $$

and then rank the candidate proteins according to their scores. This corresponds to a scoring model in which the contribution of individual proteins is assumed to be independent, implying that the probability of association of protein *q* with all proteins in *P* can be written as $1 - \prod _{p \in P} (1-S_{p,q})$. The STRING protein alias file was used to map UniProt accession numbers to Ensembl protein IDs. Results were compared with those obtained using the Endeavour prioritization software [19,20]. Endeavour also ranks a given candidate based on its similarity to a training set. However, in this case ranks are determined for each data source separately and then fused into a global ranking using order statistics. Since Endeavour is gene-based, we first mapped UniProt accession numbers to Ensembl gene IDs using the Bioconductor package *biomaRt*. Endeavour was used in batch mode.

**5. Gene expression-based filtering.** As an optional step we filter ranked candidates lists via the Gene Expression Barcode 3.0 [15], which dichotomizes gene expression data in expressed and unexpressed genes on a per sample basis. For this purpose we selected a microarray experiment with a setup similar to the 2D-DIGE experiment, comparing CD4^+^ T-cells of 11 HIV^+^ individuals and 9 HIV^-^ control individuals (GEO accession number: GSE9927, platform: Affymetrix Human Genome U133 Plus 2.0). We used functions *frma* and *barcode* from the Bioconductor package *frma* to determine the gene expression barcode for this dataset. Probeset identifiers were mapped to UniProt accession numbers using Bioconductor packages *biomaRt* and *hgu133plus2.db*. A protein was excluded from the candidate list if and only if all corresponding probesets were expressed on none of the selected microarrays. Proteins without corresponding probeset identifier were not excluded.

### Evaluation

We evaluated the performance of our prioritization method by leave-one-out cross-validation (LOOCV). This involves repeatedly leaving out a single spot from the seed list of 105 PMF-identified spots and considering it as unidentified in order to evaluate performance of our prioritization method. From the in-gel (*x*,*y*) coordinates of the excluded spot a list of candidate proteins was prioritized using the approach described above. This means in particular that the smoothing splines were refit for each cross-validation fold. We then determined the rank of the protein corresponding to each excluded spot and report the true-positive rate (TPR), that is the fraction of spots for which the correct protein appeared among the top *n* candidates for *n*∈{5,10,15,25}. True-positive rates were determined for all combinations of values *Δ*∈{0.04,0.08,0.12,…,1} for the absolute difference from the estimated pI and *δ*∈{1,2,3,…,30*%*} for the range around the estimated Mw.

### Implementation

The prioritization approach has been implemented in the statistical software package R (v3.0.2). STRING version 9.1 protein links and protein aliases files were downloaded from the STRING website [21]. Files were loaded into an in-house PostgreSQL database. The STRING payload mechanism was accessed using the Bioconductor package *STRINGdb*. R scripts and data files are available as Additional file 2.

## Results

### Influence of pI and Mw range

**Figure 2:**
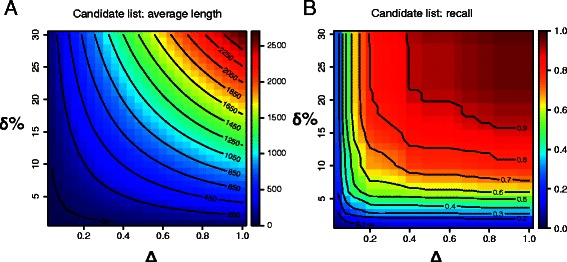
**Influence of pI range (** ***Δ*** **) and Mw range (** ***δ*** **) specified for TagIdent.** **(A)** Influence on the average number of proteins in the candidate list. **(B)** Influence on recall, that is the fraction of seed proteins included in their own candidate list as returned by TagIdent. For each identified spot in the 2D-DIGE dataset and all combinations of predefined values for the pI and Mw range, a candidate list was generated following Steps 1–3 of our prioritization approach using LOOCV.

An important ingredient of our prioritization approach is the information provided by the (*x*,*y*) coordinates of an unidentified spot on the pI and Mw of the protein(s) that migrated there. However, this information is noisy and can lead to considerable differences between observed and predicted pI and Mw values (Figure 1, Step 2). Such differences can, for example, be caused by undetected posttranslational modifications (PTMs) leading to changes in migration behaviour in both dimensions, as PTMs can alter both overall apparent molecular mass and charge. All SDS-PAGE separation techniques also have hydrophobic proteins showing anomalous migration due to extra SDS binding [22]. These factors and others can lead to errors of more than 10% when using SDS-PAGE to determine the Mw of a protein [23]. Our method takes the uncertainty of the predicted pI and Mw values into account and generates a list of candidate proteins for an unidentified spot using TagIdent by specifying the pI and Mw range around the estimated pI and Mw values (Figure 1, Step 3). The size of the chosen pI and Mw range is has a large influence on the performance of the prioritization method. When choosing ranges too narrow, candidate lists become short and the probability of the correct protein being included is small (Figure 2). For the smallest pI range (*Δ*=0.04) and Mw range (*δ*=1*%*), the average number of proteins in a candidate list was 6 with a recall of 6%. When choosing ranges too large, candidate lists in general contain the correct protein but become very long. For the largest pI range (*Δ*=1) and Mw range (*δ*=30*%*), the average number of proteins in a candidate list was 2,626 and 96.2*%* of the seed proteins appeared in their own candidate list. However, long candidate lists will likely lead to the correct protein being lowly ranked after prioritization. With a more moderate choice of pI and Mw range, for example *Δ*=0.2 and *δ*=8*%*, 60.9% of the seed proteins were contained in their own candidate list with an average candidate list length of 199.

### Prioritization performance

To prioritize candidate lists that can contain hundreds of proteins, we assumed that the proteins differentially expressed in the 2D-DIGE dataset were functionally related and thus not distributed randomly on a protein association network. The list of 92 PMF-identified proteins was indeed enriched in STRING interactions, with 377 observed interactions as compared to 66.2 expected interactions (*P*<0.0001, [18]). We, therefore, prioritized candidate proteins based on their similarity to the set of PMF-identified proteins (Figure 1, Step 4).

**Figure 3:**
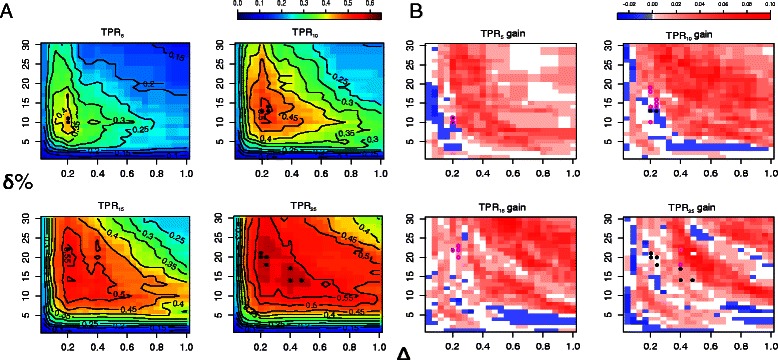
**Prioritization performance.** **(A)** True-positive rates TPR_*n*_ for the top *n*=5,10,15,25 ranked candidates using Steps 1–4 of our prioritization method for all combinations of predefined values for the pI range (*Δ*) and Mw range (*δ*). **(B)** Gain (red) or loss (blue) in TPR w.r.t. the TPR reported in panel **(A)** when also performing gene expression-based filtering (Step 5). Combinations of pI and Mw range for which the TPR reaches its maximal value are indicated with solid black dots (without filtering) and open magenta dots (with filtering).

We applied our prioritization method to the seed proteins using LOOCV. For each combination of predefined values for the pI and Mw range, the true-positive rate was calculated as a measure of performance. The TPR_*n*_ for the top *n*=5,10,15,25 ranked proteins of the candidate list is shown in Figure 3A. The maximal value for TPR_5_ equaled 0.438 for ranges *Δ*=0.2 and *δ*∈{10,11}*%*. This means that 43.8% of the seed proteins were ranked in the top-5 using our approach. For higher values of *n*, the TPR increased with a maximal TPR_25_= 0.6. As hypothesized in the previous section, large pI and Mw ranges led to inferior performance with a low TPR in the upper right corner of the contour plots (Figure 3A). Performance increased when reducing either of the ranges and then decreased again for even narrower ranges.

Results presented in Figure 3A were based on the combined STRING association scores computed by integrating the probabilities from seven different evidence types. We assessed the contribution of each individual evidence type by calculating type-specific TPR values when ranking the candidate proteins. ‘Gene coexpression’ and ‘textmining’ contributed most to the overall ranking with a maximal TPR_5_ equal to 0.41 and 0.371, respectively (Table 1). ‘Gene fusion’ only had a very minor contribution with TPR_5_= 0.076. The superior performance for ‘gene coexpression’ and ‘textmining’ is explained by the fact that these evidence types have a large coverage, whereas events such as ‘gene fusion’ are relatively rare. Also for higher values of *n*, the coexpression-based and the combined association-based TPR were highly similar (Table 1). We also assessed the contribution of low-confidence asociations to the overall ranking by comparing the TPR using our current strategy, i.e. no cut-off on the STRING association score, and using a required score of 0.15, 0.4, and 0.7 respectively. Low-to-medium confidence scores positively contribute to the overall ranking, with a considerable decrease in TPR for cut-offs of 0.4 and 0.7.

**Table 1:** **Prioritization performance**

**STRING evidence type**	**Cut-off**	**TPR**_**5**_	**TPR**_**10**_	**TPR**_**15**_	**TPR**_**25**_
Neighbourhood	0	0.238	0.276	0.305	0.352
Gene fusion	0	0.076	0.095	0.114	0.181
Cooccurrence	0	0.2	0.248	0.257	0.286
Coexpression	0	0.41	0.514	**0.571**	0.61
Experiments	0	0.295	0.343	0.39	0.457
Database	0	0.229	0.295	0.343	0.39
Textmining	0	0.371	0.438	0.486	0.533
Combined	0	0.438	**0.514**	**0.571**	0.6
Combined (gene expression-based filter)	0	**0.438**	**0.514**	**0.571**	**0.629**
Combined	0.15	0.429	**0.514**	**0.571**	0.6
Combined	0.4	0.410	0.476	0.505	0.543
Combined	0.7	0.305	0.4	0.429	0.514

True-positive rates TPR_*n*_ estimated using LOOCV for the top *n* = 5, 10, 15, 25 ranked candidates using our prioritization approach with single evidence type association scores and combined association scores. STRING assocation scores with a value less than the cut-off value were not taken into account. With a cut-off value of zero all associations contribute to the overall ranking score. Maximal TPR across all combinations of predefined values for the pI and Mw range is reported. For each value of *n* the highest TPR is indicated in bold.

In human cells, transcription has been reported to explain only 30% of variation in protein abundance levels, with translation and protein degradation contributing up to 40%. However, mRNA abundance is often a very good indicator whether or not the corresponding protein is detectable [24]. Thus, pruning a candidate list by filtering out proteins for which the corresponding gene is not expressed in a microarray experiment performed under similar conditions might eliminate unlikely candidates. We used the Gene Expression Barcode [15] to determine presence and absence calls from a microarray experiment comparing gene expression in CD4^+^ T-cells of HIV^+^ individuals and HIV^-^ control individuals (Figure 1, Step 5). Of the 91 PMF-identified proteins that could be mapped to a probeset identifier, 81 showed evidence of expression at the mRNA level. Thus, the seed proteins were indeed strongly enriched for expression at the mRNA level; of the 23,366 human proteins that could be mapped to a probeset ID, only 7,335 showed evidence of mRNA expression (*P*<2.2·10^−16^, Fisher’s exact test). Using gene expression-based filtering, we observed no increase in the maximal TPR_5_ and only a slight improvement in the maximal TPR_25_ with a 5% increase from 0.6 to 0.629 (Table 1 and Additional file 3). Note, however, that for almost all combinations of predefined values for the pI and Mw range, the TPR after filtering is at least as high as before filtering (Figure 3B).

We also compared our results with those obtained using Endeavour [19] to prioritize candidate proteins (Step 4). Endeavour is a popular prioritization tool that compared favorably to most other tools in a recent benchmarking study [25]. Endeavour uses multiple heterogeneous data sources to rank the proteins in the candidate list. We selected GeneOntology, Kegg, IntAct, String, Text and Blast as data sources. Maximal TPR values using Endeavour were considerably lower with TPR_5_= 0.324 and TPR_25_= 0.581 (Additional file 4). Possibly, the drop in performance using Endeavour is related to its use of an older STRING version.

### Prioritization of unidentified spots

We applied our prioritization method to the 188 unidentified, differentially expressed spots from the 2D-DIGE dataset (Additional file 5). Based on the LOOCV results mentioned earlier, *Δ*=0.2 and *δ*=11*%* were chosen as pI and Mw range for TagIdent leading to an average candidate list length of 242. Using gene expression-based filtering we obtained a total of 393 unique proteins that were included in at least one top-5. As an in silico validation, we examined whether these top-5 candidate proteins had a documented relationship with HIV-1 infection. For this purpose we used the NIAID HIV database of (HIV-1)–human protein interactions [26]. Of the 389 unique top-5 candidate proteins that could be mapped to an Entrez Gene ID, 213 had documented evidence for interactions with HIV-1 proteins. Thus, the top-5 candidates were strongly enriched for such interactions; of the 12,544 proteins found in at least one candidate list, only 1,659 showed evidence of interactions with HIV-1 proteins (*P*<2.2·10^−16^, Fisher’s exact test). The efficacy of our strategy is also illustrated by a clear decreasing trend in the occurrence of HIV-1 interacting proteins at lower ranks (Additional file 6). This provides strong evidence that our prioritization method provides candidate proteins that are plausible in terms of their in-gel migration behavior and functional relevance.

## Discussion

Although the human proteome can now be probed at an unprecedented scale [27], the identification and quantification of low-abundant proteins remains a formidable challenge. We presented a prioritization method that generates ranked lists of candidate proteins for unidentified low-abundant (i.e. only visible using fluorescense) spots from a 2D-DIGE experiment. Candidate proteins are proposed, based on the in-gel location of a spot, and resulting candidate lists are ranked, based on the strength of association of candidates with the PMF-identified proteins using STRING functional association scores. We assessed the performance of our approach on proteins differentially expressed at the peak of HIV-1 infection of T-cells [6]. Evaluation by LOOCV showed that our method ranked 43.8% of the proteins in the top-5 of their respective candidate lists.

**Figure 4:**
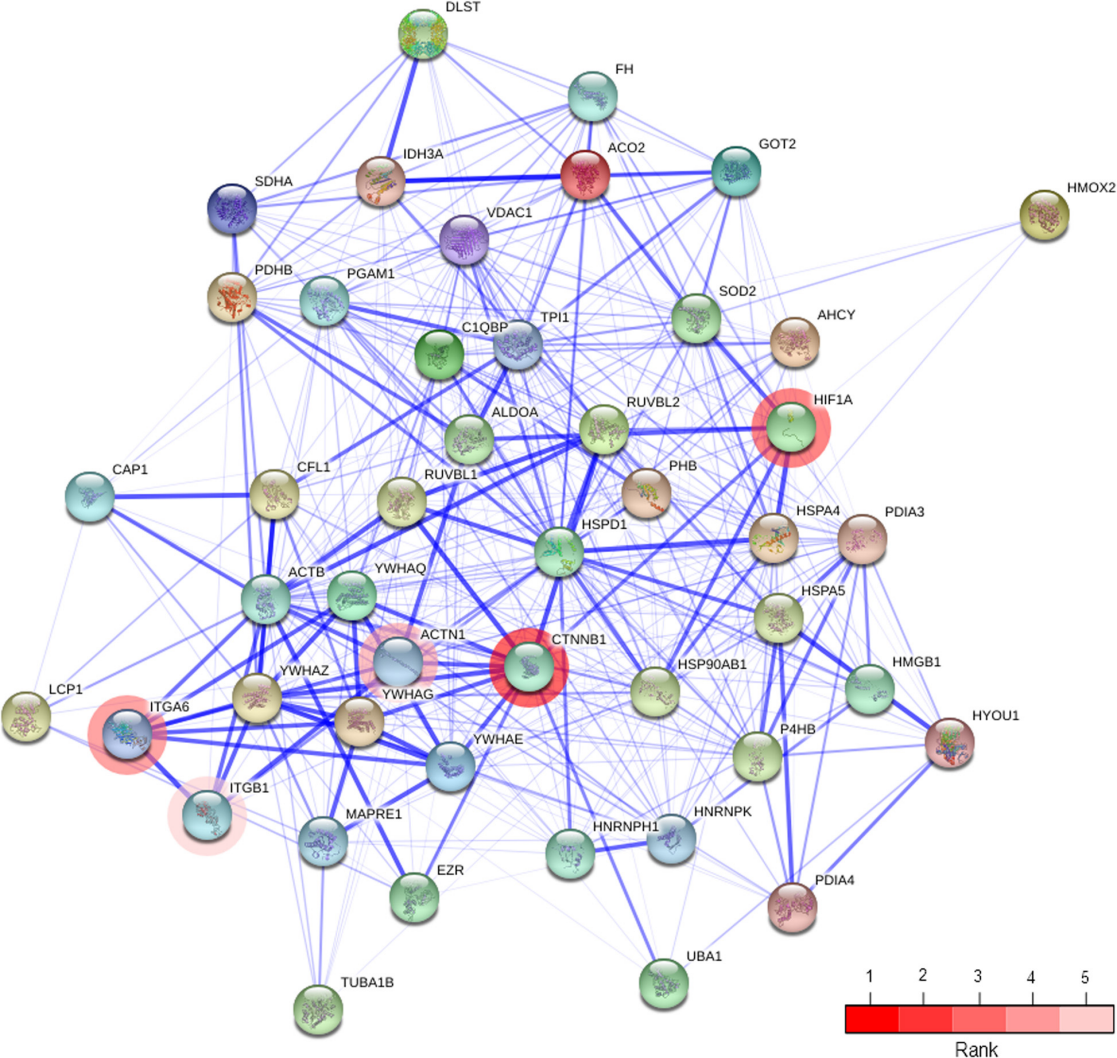
**STRING-based visualization of candidate proteins.** Visualization of the top-5 candidate proteins for unidentified spot (*x*,*y*)=(669,201) and the seed proteins directly connected to them in STRING. Candidates 1–5 are shown with red highlights of decreasing intensity, highlighted using STRING’s payload mechanism. Connections between proteins indicate the confidence for an association, stronger associations are represented by thicker lines.

Several other approaches have been developed to prioritize genes and - to a lesser extent - proteins from a list of candidates based on text mining, similarity profiling and network analysis [28]. Existing tools have several limitations for our purpose. First, most tools are web-based and do not provide a programmatic interface or allow for batch queries. They are therefore not suited for our experimental setup, which involves leave-one-out cross-validation on 92 proteins for 25·30=750 combinations of possible values for the pI and Mw range. Second, prioritization methods often integrate multiple data sources and are therefore difficult to keep up-to-date. Third, surprisingly, most prioritization tools do not provide detailed information about the evidence on which the candidate ranking is based [28]. Such information is invaluable for making an informed decision on which top candidates to validate experimentally. We decided to base our prioritization method on functional protein-protein associations provided by STRING. Over the past 10 years STRING has established itself as a high-quality resource of functional links between proteins. Moreover, the data content of STRING is frequently updated and all information regarding interactions and the interacting proteins themselves can be downloaded. This enabled us to develop a computational pipeline that can be easily updated to a new of version of STRING. One of the main strengths of STRING is its interactive and intuitive user interface, which provides a detailed overview of the STRING network and the evidence for each protein-protein association. We employed the payload mechanism that enables projecting external information onto STRING [18] to visualize networks consisting of seed proteins and top-5 candidate proteins for unidentified spots (for a typical example, see Figure 4; for the full list, see Additional file 5). In addition, we integrated information on the rank of a candidate protein and on evidence of (HIV-1)–human protein interactions for each of the top-5 candidates to further enhance interpretation (Additional file 7). We also demonstrated that our prioritization method clearly outperformed Endeavour. However, prioritization could possibly be further improved by also incorporating association scores of indirect interactions, for example via prioritization based on random walks or diffusion kernels [29].

Despite the capability of our approach to correctly rank the correct protein among the top candidates, it has several potential limitations. First, we could not take the extensive diversification of the human proteome due to different isoforms, posttranslational modifications and processing (e.g. of signal peptides) into account. In order to determine the theoretical pI and Mw of the identified proteins we assumed that their spots corresponded to the most abundant, mature form. Clearly, the estimated calibration curves are affected by the validity of this assumption. This problem is illustrated by the fact that multiple spots corresponding to different forms of the same protein had to be assigned identical pI and Mw values; see for example the four spots for heat shock protein 60 (P10809; Additional file 1). This partly explains the relative inaccuracy of the predicted pI and Mw values and the rather wide optimal pI and Mw ranges *Δ*=0.2 and *δ*∈{10,11}*%*. These wide ranges presumably also lead to several proteins being highly ranked for multiple unidentified spots (Additional file 5). For example, catenin beta-1 (P35222; pI=5.53, Mw=85365) was ranked first for 8 spots with predicted pI values in the range 5.34–5.42 and predicted Mw values in the range 82351–95847. Whether these spots really represent (modified forms of) catenin beta-1 awaits further experimental validation. Note that even if CTNB1 is not in any of these spots, it can still be differentially expressed in our experiment, as it is strongly associated with the already identified differentially expressed proteins [9]. Isoforms are also not taken into account by STRING, only the mature form being used in the prioritization step. However, TagIdent contains all isoforms listed by UniProt even though some of them must be really rare. This implies that for spots corresponding to non-canonical isoforms, correct proteins can still end up in the candidate list. For example, cellular tumour antigen p53 (P04637; pI = 6.33, Mw = 43653), for which UniProt lists 9 isoforms, was ranked first for 11 unidentified spots with predicted pI values in the range 5.34–7.91 and predicted Mw values in the range 31284–45252. One should also remember that the optimal ranges for pI and Mw are determined by a trade-off between specificity and sensitivity. With a pI deviation +/-0.2 and an Mw deviation +/-11%, the average number of proteins in a candidate list is 266 and 67.6% of the seed proteins appear in their own candidate list. Thus, even with optimal ranges almost one-third of the seed proteins could not be prioritized since differences between observed and predicted pI or Mw values were too large (Additional file 1). Some deviations are probably caused by lacking data for extreme values of *x* or *y* coordinates. For example, pI deviations are large for such highly basic proteins as 40S ribosomal protein S5 (P46783) and Histone H2B type 1-L (Q99980). However, large deviations can also be observed for intermediate values of the *x* or *y* coordinates. For example, triosephosphate isomerase (P60174) displayed both a pI deviation of -1.113 and an Mw deviation of 22.9%. Differences such as these occur if the corresponding spot did not contain the mature form of the protein or, more likely, was posttranslationally modified.

With 188 unidentified spots a lot of candidate lists are being generated. Even looking at the top-5 lists only, 940 candidates could in principle come up. The actual number is much less, 393 unique proteins, as many candidates come up multiple times (e.g. P53; 11 times or HIF1A (Q16665); 17 times). Still, it is easy to cherry-pick some proteins from these lists for discussion of their possible roles in the context of HIV–T cell interaction. Checking whether a candidate is indeed differentially expressed should be performed first, e.g. by Western blotting. Working out whether the expression pattern change is due to PTMs is harder, and working out the biological relevance of the change is harder still. Keeping in mind these caveats, the chances that real changes are occurring in the 2D patterns of e.g. the two predicted proteins mentioned, P53 and HIF1A, are rather good. P53 is a protein that strongly reacts to cellular stresses both at the level of amounts and PTM changes, which, as a very central player, is involved in regulating choices between cell growth, cell arrest for repair, or apoptosis. HIF1A, also a central switch protein, is, amongst others, involved in the choice between more pronounced glycolysis with less oxidative phosphorylation and ‘normal’ glycolysis with more pronounced mitochondrial oxidative processes. This so-called Warburg shift can occur in T-cells [30], and is heavily influenced by HIF1A. Interestingly, the identified proteins from our dataset (Additional file 1) showed a clear down regulation of proteins involved in glycolysis [6]. An overall down regulation of HIF1A, as it stimulates glycolysis, would thus be expected. However, the difficulty of making sense of PTM patterns is nicely illustrated in this case: HIF1A pops up 17 times in the candidate top-5 lists, 9 times in spots that are up regulated in response to infection, 8 times in down regulated ones. How many of these spots really represent HIF1A and in what forms remains to be investigated, but HIF1A is clearly one of the candidates that deserve further study, nicely illustrating the power of our approach. The information regarding up and down regulation of specifically modified forms of such important proteins can be obtained much more efficiently from large-scale 2D-DIGE experiments with the aid of our computational method. Small amounts of protein from both control and infected T-cells could be run on small IEF strips having the appropriate (restricted) pI range, followed by standard SDS-PAGE separation. Upon Western blot analysis, specific protein patterns will be obtained. Combining these with the more reliable quantitative information in the original 2D-DIGE experiments could illuminate how the protein of interest has been modified in response to the stimulus under investigation, in this case viral infection. In conclusion, though using 2D-DIGE datasets in combination with our algorithm to analyse changes in PTMs upon a biological stimulus is not straightforward, it represents a promising alternative to study this crucial way of responding to changes in the environment.

The applicability of our method could be extended in several ways. First, in 2D-DIGE experiments with only limited amounts of differentially expressed spots one could identify additional, non-differentially expressed spots using mass spectrometry to fit more reliable calibration curves. Secondly, it is conceivable that certain affected cellular pathways are not represented in the set of abundant identified seed proteins used in our prioritization approach. Although more difficult, one could look at pathway clustering in the ranked candidate lists directly. This might lead to the unbiased identification of interrelated low-abundant protein changes. Finally, iteratively improving the analysis should be straightforward: whenever further analysis based on the respective candidate lists gives additional identified proteins, both calibration curves and the prioritization approach for candidate ranking can be further optimized.

## Conclusions

The combination of 2D-DIGE experiments and the prioritization approach proposed in this paper provides a verstatile technique able to identify differential proteins in the lower regions of the dynamic range. We expect it to be useful in (re-)mining other 2D-DIGE experiments in which especially the low-abundant (not Coomassie stainable) protein spots remain to be identified. Candidate proteins prioritized by our approach are good candidates for further study and will surely contribute to a better understanding of the biological mechanisms studied in 2D-DIGE experiments.

## Additional files

Additional file 1
**List of PMF-identified proteins and their characteristics.** Updated and extended version of Table S1 in Ringrose et al., J Virol. 2008 May;82(9):4320-30.

Additional file 2
**R scripts and data files used in calculating the results presented in the manuscript.**

Additional file 3
**Prioritization performance using gene expression-based filtering.** True-positive rates TPR_*n*_ estimated using LOOCV for the top *n*=5,10,15,25 ranked candidates using Steps 1–5 (including gene expression-based filtering) of our prioritization method for all combinations of predefined values for the pI range (*Δ*) and Mw range (*δ*).

Additional file 4
**Prioritization performance (Endeavour).** True-positive rates TPR_*n*_ estimated using LOOCV for the top *n*=5,10,15,25 ranked candidates using Endeavour to prioritize candidate proteins for all combinations of predefined values for the pI range (*Δ*) and Mw range (*δ*).

Additional file 5
**List of top candidate proteins for unidentified spots.** Overview of the top candidate proteins for the 188 unidentified, differentially expressed spots from the 2D-DIGE dataset. Also provides links to STRING-based visualizations of the seed proteins and the top-5 candidate proteins generated using STRING’s payload mechanism.

Additional file 6
**Fraction of HIV-1 interacting proteins.** For each rank in the candidate lists of the 188 unidentified proteins, the average fraction of proteins with documented evidence for interactions with HIV-1 proteins in the NIAID HIV database of (HIV-1)–human protein interactions is displayed.

Additional file 7
**STRING-based visualization of candidate proteins.** Visualization of the top-5 candidate proteins for unidentified spot (*x*,*y*)=(669,201) and the seed proteins directly connected to them in STRING. Candidates 1–5 are shown with red highlights of decreasing intensity, highlighted using STRING’s payload mechanism. Connections between proteins indicate the confidence for an association, stronger associations are represented by thicker lines. Pop-ups show integrated information on the rank of HIF1A in the candidate list and its reported interactions with HIV-1 proteins.
