# Supplementary figures and images for: Candidate prioritization for low-abundant differentially expressed proteins in 2D-DIGE datasets
Source: BMC Bioinformatics. 2015 Jan 28;16:25. doi: 10.1186/s12859-015-0455-x (PMC4384356; doi:10.1186/s12859-015-0455-x)

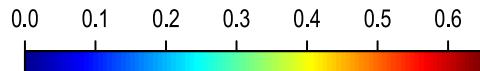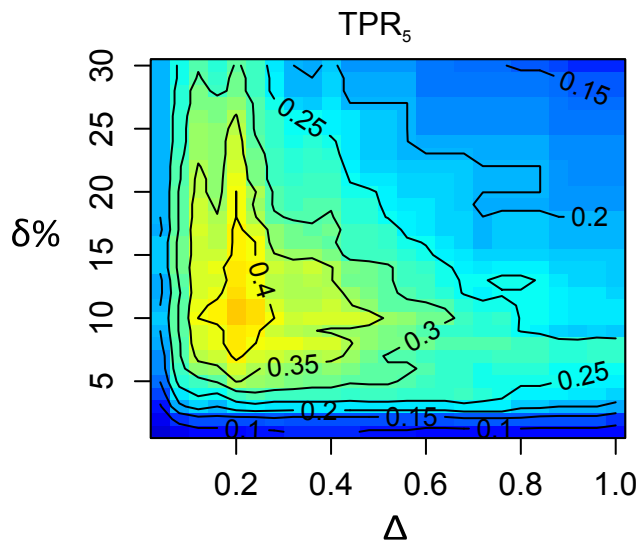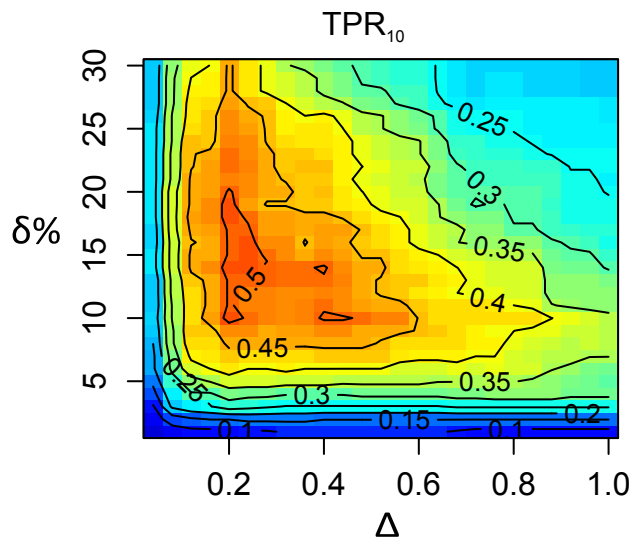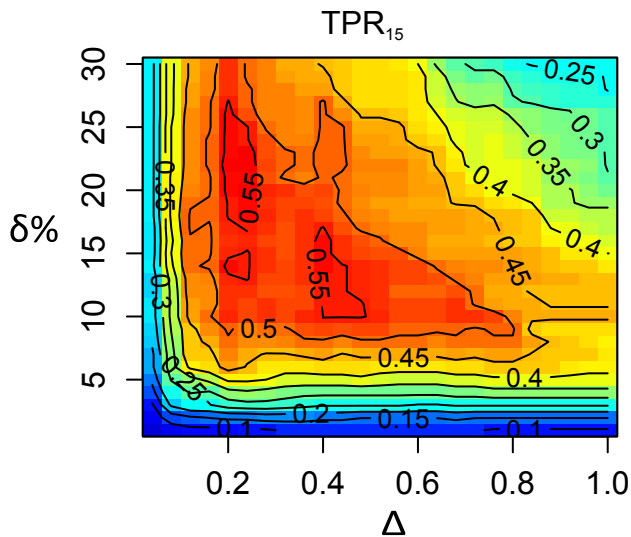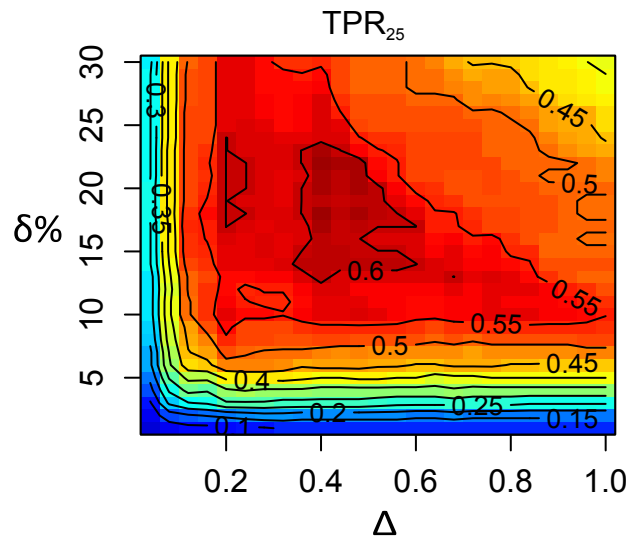

Supplement: Additional file 3 — Prioritization performance using gene expression-based filtering. True-positive rates TPRn estimated using LOOCV for the top n=5,10,15,25 ranked candidates using Steps 1–5 (including gene expression-based filtering) of our prioritization method for all combinations of predefined values for the pI range (Δ) and Mw range (δ). [file 12859_2015_455_MOESM3_ESM.pdf]

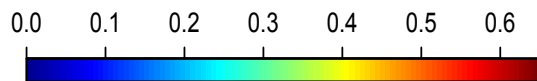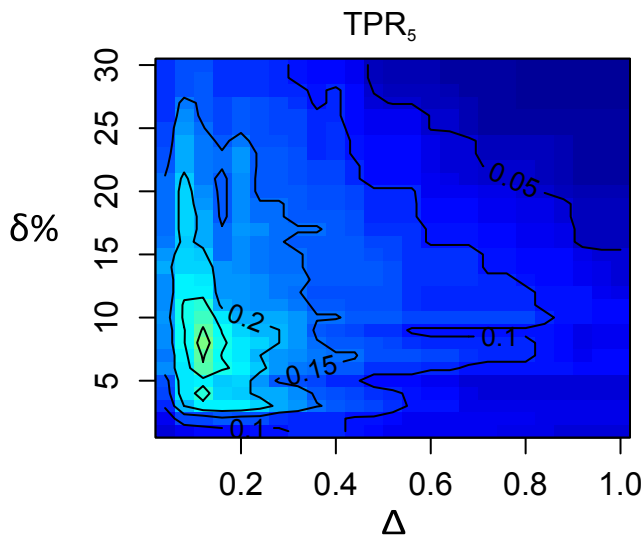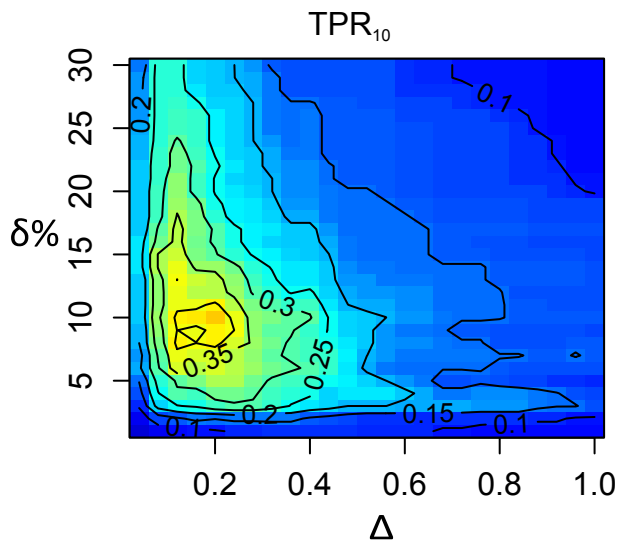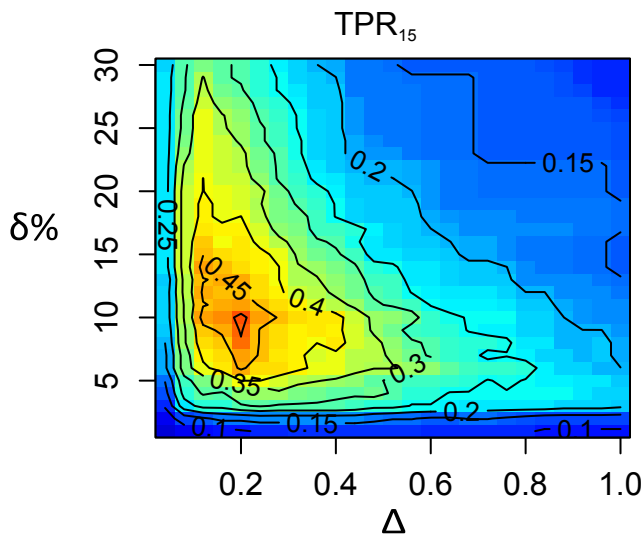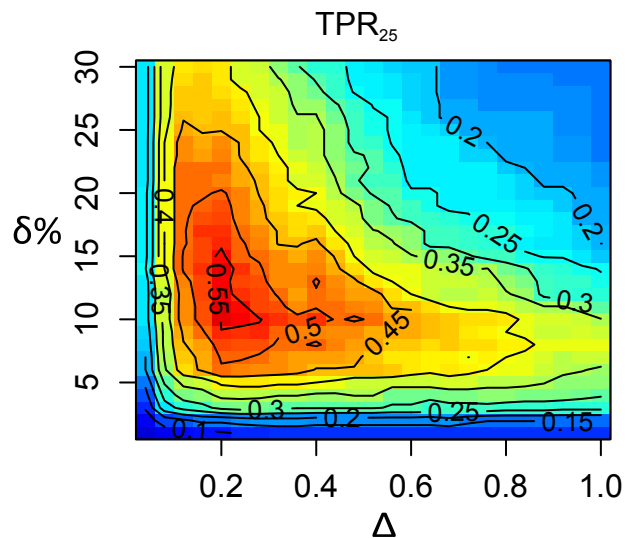

Supplement: Additional file 4 — Prioritization performance (Endeavour). True-positive rates TPRn estimated using LOOCV for the top n=5,10,15,25 ranked candidates using Endeavour to prioritize candidate proteins for all combinations of predefined values for the pI range (Δ) and Mw range (δ). [file 12859_2015_455_MOESM4_ESM.pdf]

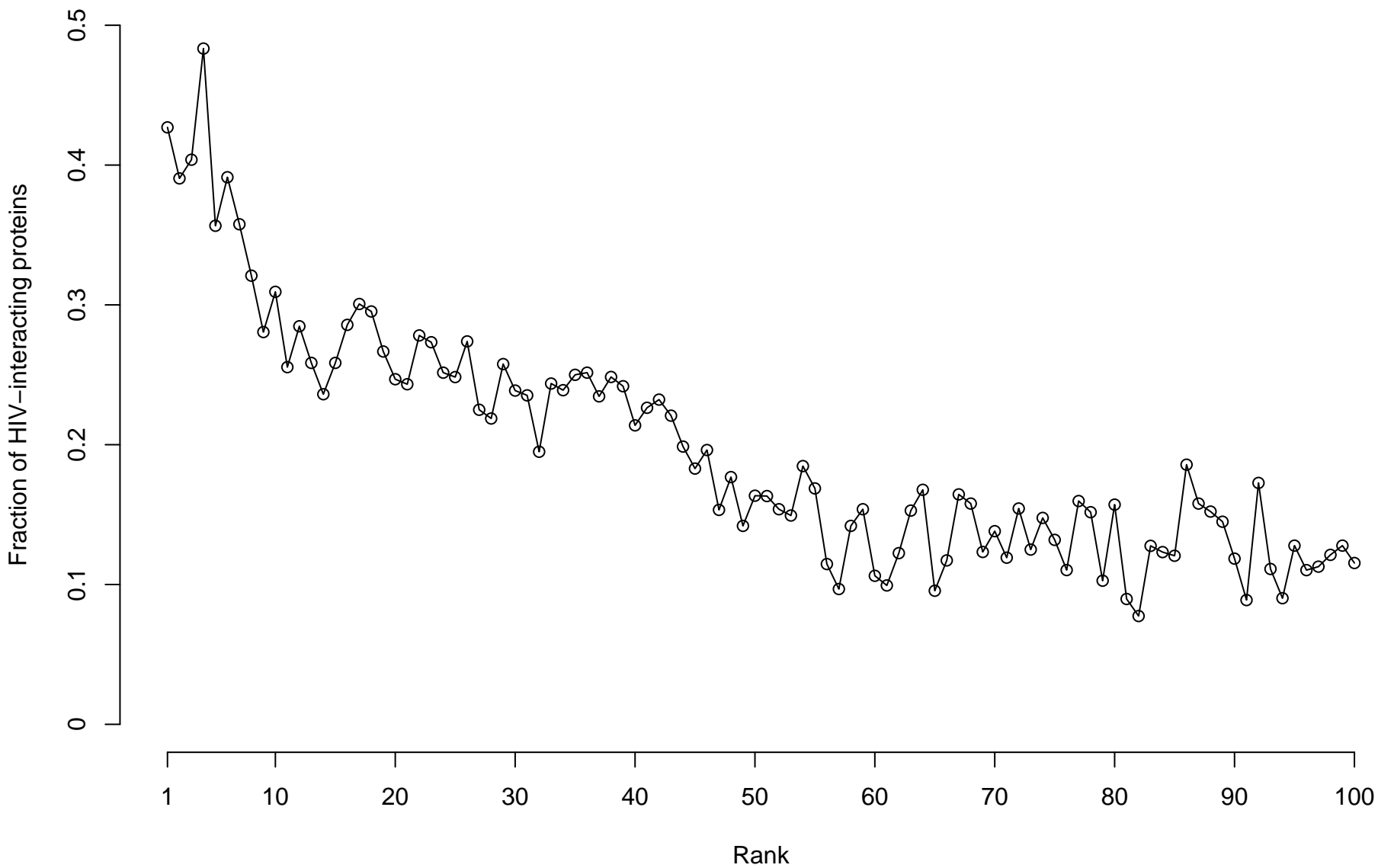

Supplement: Additional file 6 — Fraction of HIV-1 interacting proteins. For each rank in the candidate lists of the 188 unidentified proteins, the average fraction of proteins with documented evidence for interactions with HIV-1 proteins in the NIAID HIV database of (HIV-1)–human protein interactions is displayed. [file 12859_2015_455_MOESM6_ESM.pdf]

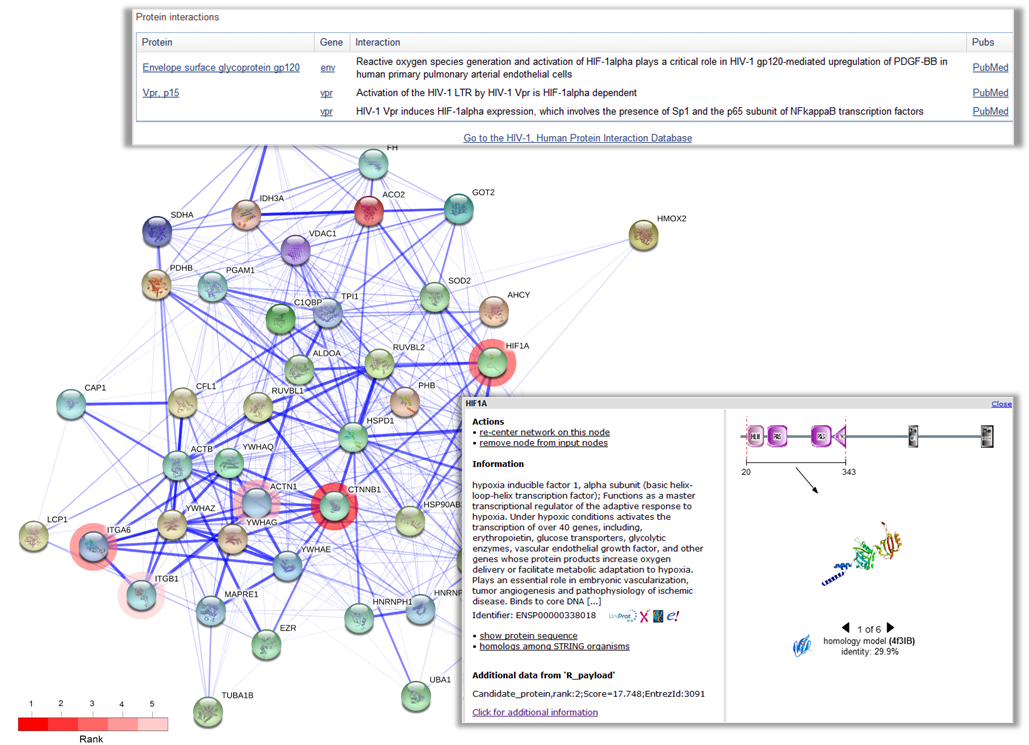

Supplement: Additional file 7 — STRING-based visualization of candidate proteins. Visualization of the top-5 candidate proteins for unidentified spot (x,y)=(669,201) and the seed proteins directly connected to them in STRING. Candidates 1–5 are shown with red highlights of decreasing intensity, highlighted using STRING’s payload mechanism. Connections between proteins indicate the confidence for an association, stronger associations are represented by thicker lines. Pop-ups show integrated information on the rank of HIF1A in the candidate list and its reported interactions with HIV-1 proteins. [file 12859_2015_455_MOESM7_ESM.png]
